# “It is the One Thing that has Worked”: facilitators and barriers to switching to nicotine salt pod system e-cigarettes among African American and Latinx people who smoke: a content analysis

**DOI:** 10.1186/s12954-021-00543-y

**Published:** 2021-09-16

**Authors:** Kim Pulvers, Myra Rice, Jasjit S. Ahluwalia, Michael J. Arnold, Crystal Marez, Nicole L. Nollen

**Affiliations:** 1 Department of Psychology, California State University San Marcos, 333 S. Twin Oaks Valley Rd., San Marcos, CA 92096 USA; 2 Department of Behavioral and Social Sciences and the Center for Alcohol and Addiction Studies, School of Public Health, Department of Medicine and Brown Cancer Center, Alpert Medical School, Brown University, Providence, RI USA; 3 Department of Population Health, University of Kansas School of Medicine, Kansas City, KS USA; 4 Neuroscience Interdepartmental Program, University of California, Los Angeles, CA 90095 USA

**Keywords:** Electronic nicotine delivery systems, Smokers, Minority groups, Tobacco products, Harm reduction, Ethnic groups, Smoking reduction

## Abstract

**Background:**

Electronic cigarettes are a harm reduction strategy for individuals who smoke cigarettes who cannot or do not want to quit using FDA-approved cessation methods. Identifying perceived facilitators and barriers to switching among people who smoke cigarettes is critical to optimizing health impact. This is particularly important for the most dominant e-cigarette device, nicotine salt pod electronic cigarettes. We investigate the experience using pod electronic cigarettes among African American and Latinx individuals who smoke, the two largest racial/ethnic minority groups who experience significant health disparities.

**Methods:**

From July 2018 to May 2019, adults who smoked cigarettes, age 21 + (*N* = 114; M age = 44.6, 59.6% male, 52.6% African American from Kansas City, 47.4% Latinx from San Diego) received JUUL-brand electronic cigarettes (referred to hereafter as JUUL) for 6 weeks and answered interview questions at week six. We inquired what they liked and disliked about using JUUL, what helped with switching and made switching difficult, future intentions for continued JUUL use, and how JUUL compared to past smoking reduction methods. Responses were coded into themes by independent raters. Theme frequencies were analyzed separately by race/ethnicity and week 6 use trajectory (exclusive JUUL use, dual JUUL and cigarette use, exclusive cigarette use).

**Results:**

Clean/smell was the aspect of using JUUL most commonly liked (23%), followed by convenience (19%). Coughing/harshness was a more common barrier to switching for African American (44%) than Latinx (9%), and for continuing cigarette use (56%) than for those who exclusively switched or dually used JUUL and combustible cigarettes (15–21%). Most (78% African American; 90% Latinx) reported that the benefits of using JUUL outweighed barriers, and this varied by JUUL use trajectory: 94% exclusive switch, 86% dual use, and 42% continued cigarette use. The majority said they would continue using JUUL to replace cigarettes (83% African American; 94% Latinx) and that JUUL worked better than other methods to reduce cigarettes (72%).

**Conclusion:**

African American and Latinx individuals who smoked experience using pod electronic cigarettes was generally positive. Understanding facilitators and impediments to switching to electronic cigarettes among racial/ethnic minority people who smoke can inform harm reduction interventions and reduce tobacco-related health disparities.

*Trial Registration* ClinicalTrials.gov Identifier: NCT03511001 posted April 27, 2018.

**Supplementary Information:**

The online version contains supplementary material available at 10.1186/s12954-021-00543-y.

## Background

Electronic cigarettes (ECs) have emerged as a harm reduction strategy for individuals who smoke cigarettes who cannot or do not want to quit using FDA-approved cessation methods, such as nicotine replacement therapy or medication [1]. An exclusive switch from cigarettes to ECs optimizes harm reduction [2–7] and there is some evidence that dual use reduces cigarette consumption and related harm [4, 5, 8]. However, African American (AA) and Latinx (Lx) people who smoke cigarettes bear a disproportionate burden of smoking-related morbidity and mortality [9, 10], experience a gap in access to tobacco treatment [9, 10], and are less likely than non-Hispanic white individuals who smoke to switch to ECs and to use them exclusively [11]. Few experimental investigations of people who smoke cigarettes using ECs for harm reduction or cessation have had adequate representation of AA and Lx individuals who smoke to provide insight into facilitators and barriers to EC use [1, 12].

Racial and ethnic disparities in exclusive switching to ECs could exacerbate the burden of tobacco-related death and disease among disadvantaged populations [13]. A previous prospective qualitative study of AA people who smoked menthol cigarettes switching to first generation cigalikes found that the majority (73%) reported an unsatisfactory experience [14]. More information on the experience of ECs among AA and Lx individuals who smoke is needed to evaluate the viability of ECs as a harm reduction strategy. This information is particularly needed for the most recent fourth generation, nicotine salt pod system (NSPS) ECs [12].

High nicotine delivery and other reinforcing features of fourth generation NSPS ECs have contributed to their market dominance [15, 16]. The same features that have led to abuse liability among adolescents [17, 18] may also support switching and potential harm reduction among adults who smoke combustible cigarettes. The present study investigates the experience of switching from smoking to a NSPS EC among adult members of the two largest racial/ethnic minority groups in the U.S., AA and Lx adults who smoke. Given the nicotine delivery profile of NSPS ECs, the experience was expected to be more positive than that of earlier generation products. However, given the paucity of EC research with racial/ethnic minority individuals who smoke and with NSPS ECs, the research aims were to describe the perceived facilitators and barriers to switching and utility of ECs compared to other methods of cigarette reduction. Furthermore, we evaluated trends in experience by EC use trajectory (exclusive EC, dual EC-cigarette, exclusive cigarette).

## Methods

This is a secondary analysis from the first reported randomized clinical trial of NSPS ECs testing effects on toxicant exposure among AA and Lx adults who smoke [19]. As described in detail elsewhere [19], this multi-site study was conducted from July 2018 to May 2019. The study was approved by the Institutional Review Board at California State University San Marcos and the University of Kansas School of Medicine and all participants provided written informed consent. Participants (*N* = 187) were randomly assigned in a 2:1 ratio, stratified by study site (AA in Kansas City, MO and Lx in San Diego, CA), to EC or cigarettes as usual. Those randomized to EC received a JUUL EC and pods in a choice of Virginia tobacco, classic menthol, cool mint, or mango flavor pods (5% nicotine) for six weeks, along with brief education, training, and action planning for making a complete switch to ECs. As reported in Pulvers et al. (2020), menthol was the leading flavor selected in the study (35.2% at baseline and 34.5% at week two). This was driven by a majority of African American participants choosing menthol (61.3% at baseline and 60.3% at week two), compared to Latinx participants choosing menthol (9.5% at baseline and 7.3% at week two).

JUUL was chosen for the study because it utilized the most recent technology available, combining the safety and simplicity of first generation products with the effective nicotine delivery of third generation products. Of the fourth generation products on the US market, JUUL was highly rated by consumers and recommended to the PI by leading tobacco regulatory scientists. JUUL uses nicotine salt instead of free-base nicotine, which replicates the efficient nicotine delivery of cigarettes using a small volume of e-liquid (0.7 mL per pod) and a low-yield battery [20]. This allows for a small, lightweight device and factory-sealed pods. The JUUL product promised to address two major barriers for people facing an exclusive switch from smoking to ECs: convenience and delivery of adequate nicotine [7].

The present study focuses on those in the final analytic sample of the EC group who completed an exit assessment at week six (114/125 = 91.2%; 60 AA; 54 Lx). Study eligibility criteria, study recruitment, and procedures for the one-time exit assessment at week six were the same as for the parent trial [19]. The exit assessment took place at the end of the week six visit. It was conducted by the same research assistant who conducted the week six visit. The week 6 visit included objective (e.g., observable, such as biomarkers measured in urine) and subjective (self-report questions) measures. All subjective measures were administered in an interviewer-assisted format, where questions were read to participants and visual cues were provided on a flip chart, (e.g., likert scales for survey questions).

### Measures

Participant characteristics were assessed in a baseline survey. These included age, sex, race/ethnicity, education, income, marital status, smoking history, current cigarette frequency, time to first cigarette, quitting history, history of respiratory illness, mental health issues, and substance abuse. Open-ended interview questions about the participant experience included what they liked and disliked about using JUUL, what helped with switching and made switching difficult, future intentions for continued JUUL use, and perception of JUUL compared to other methods to reduce smoking used in the past. A single forced choice interview question assessed the relative benefits of switching to JUUL vs. the barriers or concerns (options included benefits do not outweigh barriers or concerns; benefits and barriers are equal; or, benefits outweigh barriers or concerns). Participant responses were entered directly into REDcap, a HIPAA compliant and password-protected online database by the researcher conducting the exit assessment. Data records were audited by each site project manager for completeness.

### Analytic approach

Open-ended responses were downloaded from REDcap into Excel. Content analysis consisted of a two-step process by two independent raters: 1) identifying themes in participant responses to each question and 2) classifying each individual response into the identified themes [21]. In cases where one rater identified a theme that the other rater did not, an independent third rater judged whether to retain the theme or whether to reclassify the theme. The independent third rater resolved discrepancies in item placement by selecting one best-fitting theme.

Once coding discrepancies were reconciled by the third rater, the number of responses in each theme were counted. A percentage was calculated by dividing the number of responses classified under each theme by the number of total coded responses. For example, in San Diego (Lx) there were 82 responses to the Like question, 17 of which were coded as clean/smell. The frequency of clean/smell among the Lx sample was calculated as 17/82 = 20.7%. The practice of quantifying information from open-ended questions and analyzing the converted data with descriptive statistics is consistent with a conversion mixed methods approach [22, 23].

Although there were no site differences in the primary outcomes of the parent trial [19], we conducted our analyses by site in order to identify potentially different experiences by population (AA/Kansas City and Lx/San Diego). The frequency of themes was also analyzed by JUUL use trajectory (exclusive JUUL use, dual JUUL and cigarette use, exclusive cigarette use. For example, in San Diego, 25 of the 82 responses to the Like question were from participants who exclusively used JUUL, 46 were from participants who used both JUUL and cigarettes, and 11 were from participants who continued smoking cigarettes. The number of responses within each JUUL use trajectory group was used as the denominator for calculating proportions of theme per trajectory group. For example, individuals who exclusively switched to JUUL reported 25 responses to the Like question, 8 were coded as clean/smell, for a frequency of 8/25 = 32%. In comparison, 6 of the 46 responses from those who dually used JUUL and cigarettes were coded as clean/smell, for a frequency of 6/46 = 13.0%. Our analysis focuses on the frequency of themes in each sub-group to characterize the experience of participants in each JUUL use trajectory group.

JUUL use trajectories were reported in Pulvers et al. (2020) and were characterized based on past 7 day cigarette and JUUL consumption from timeline follow back assessment at week six and bio-verification of exclusive JUUL use (carbon monoxide < 6 parts per million) [19]. At week six, 28.1% were classified as exclusively using JUUL; 57.9% dually used JUUL and cigarettes, and 14.0% exclusively used cigarettes.

## Results

Before resolving discrepancies, the number of matching themes, defined as those that were identically-named or had similar response content, ranged from 90.9 to 100% (Table 1). In most cases, the number of themes identified per question varied slightly by population. Agreement in individual item placement into themes ranged from 73.1% to 95.5% (Table 1). Rater agreement was in the 90%-range for three themes, 80%-range for eight themes, and 70%-level for one theme. The 70%-level discrepancy reflected item placement into “practice” versus “self-regulation/motivation” theme for the question regarding what helped with switching. For example, Rater 1 placed the response “just stick with it” under “practice” while Rater 2 placed this item under “self-regulation/motivation.”

**Table 1:** Open-ended question coding and rater agreement

Item	African American/Kansas City			Latinx/San Diego		
	Rater theme agreement %	N themes	Rater item agreement %	Rater theme agreement %	N themes	Rater item agreement %
Like	100	9	83.3	100	10	86.7
Not like	100	7	95.5	91.7	8	89.8
Helped	92.9	10	73.1	100	11	85.3
Difficult	100	9	93.5	90.9	8	87.3
Continue [YES]	90.9	8	85.2	93.3	8	81.1
Comparability	100	7	93.4	90.9	9	88.1

Table displays the rater theme agreement percent, the number of themes, and the rater item agreement percent for responses to what participants liked about using JUUL, what participants did not like about using JUUL, what helped participants with switching to JUUL, what made switching to JUUL difficult, why participants would continue using JUUL to replace cigarettes, and how JUUL compare to previous cigarette reduction methods

Representative quotes illustrating themes are provided in Table 2.

**Table 2:** Representative quotes per theme

Theme	Representative quotes
*JUUL likes*	
Clean/smell	Nice not feeling like you smelled bad after, not having residue and ash all over (Lx)
Convenience	I liked that it was convenient. No lighters (AA)
Health	My breathing got better (Lx)
Cost	Saving money (AA)
Taste	The taste (Lx)
Subjective effects	I enjoyed it, I like seeing the cloud of smoke it produces, by seeing that I tell that I am getting a hit (Lx)
Cravings/cessation	It took away the urge to smoke (AA)
Nothing	Nothing (AA & Lx)
Social	It was acceptable. I didn’t have to keep a cigarette away from people (Lx)
Use anywhere^a^	That I could smoke it when I wanted wherever I wanted (Lx)
*Helped with switching*	
Self-regulation/motivation	Just sticking with it and not giving up (AA)
Nothing/NA	Nothing (AA & Lx)
Cravings/cessation	It satisfied the craving (AA)
Health	Seeing the health benefits helped me stick with it (Lx)
Convenience	Not needing a lighter (AA)
Social	Socially fun (AA)
Cost	Didn't spend as much money buying cigarettes (Lx)
Program support	This program helped me switch (AA)
Practice^b^	Learning how to puff correctly, at the correct angle (AA)
Subjective experience^b^	How clean it was, felt similar to a cigarette but cleaner (AA)
Flavor^a^	It tastes good (Lx)
Smell^a^	Not wanting to have the smell of cigarettes (Lx)
Other^a^	The e-cig itself (Lx)
*JUUL dislikes*	
Nothing	Nothing (AA & Lx)
Side effects (coughing/harshness)	Coughing/harshness in throat (AA)
Mechanical issues—Pods	Pods were leaking (Lx)
Inconvenience^b^	That I lost it a couple times (AA)
Shape^b^	The shape was weird at first (AA)
Taste^b^	Metallic taste (AA)
Learning to use^b^	Figuring out how big of a puff to take (AA)
User issues^a^	Have to remember to charge it (Lx)
Mechanical issues—battery^a^	I don’t like how they die (Lx)
Comparability^a^	I miss texture of the cigarettes (Lx)
Nicotine exposure^a^	The amount of nicotine I was smoking increased (Lx)
Cost^a^	The prices, and having to buy a four pack (Lx)
*Made switching difficult*	
Side effects	Harshness, coughing (AA)
Nothing	Nothing (AA & Lx)
Comparability	Just being used to smoking cigarettes for the past 18 years (Lx)
Other	Stress and alcohol were all factors (AA)
Taste^b^	I was not a fan of the cool mint taste at first (Lx)
Readiness to quit^b^	Wasn't mentally ready to give up cigarettes (AA)
Strength/nicotine intensity^b^	Felt like too much nicotine (AA)
Mechanical issues—pods^b^	The e-juice issue (AA)
Learning to use^b^	Had to learn how to puff correctly (AA)
User issues^a^	Losing charger (Lx)
Craving for cigarettes^a^	The urge of smoking a cigarette is there from the smell and other reminders (Lx)
Social^a^	Other people smoking regular cigarettes around me (Lx)
Cost^a^	Paying for it (Lx)

Table displays representative quotes per theme for responses to what participants liked about using ECs, what helped participants with switching to ECs, what participants did not like about using ECs, and what made switching to ECs difficult

^a^Theme is unique to Lx/San Diego sample

^b^Theme is unique to AA/Kansas City sample

Participants were composed of 59.6% male, 52.6% AA, and 47.4% Lx, and the average age was 44.6 years old. Participants smoked an average of 12.4 cigarettes per day at baseline, had been smoking for an average of 17.3 years, and about half smoked menthol cigarettes (Table 3). Past year quit attempts were of similar frequency among AA (30%) and Lx (35%) participants and there was variation in their cessation methods (Table 3). Using nicotine replacement therapy and alternate tobacco products was reported more often by Lx, and using Chantix was reported more often by AA (Table 3).

**Table 3:** Baseline study sample characteristics of e-cigarette (EC) group

Variable	All	Race/ethnicity		Tobacco use trajectory		
	Mean (SD) or N (%)	Mean (SD) or N (%)		Mean (SD) or N (%)		
	*N* = 114	African American *n* = 60	Latinx *n* = 54	Exclusive EC use *n* = 32	Dual EC and cigarette use *n* = 66	Continued cigarette use *n* = 16
Age	44.6 (12.9)	51.4 (9.4)	37.1 (12.1)	44.4 (13.8)	43.9 (13.0)	48.0 (10.6)
Sex, % female	46 (40.4)	32 (53.3)	14 (25.9)	16 (50.0)	25 (37.9)	5 (31.3)
African American	60 (52.6)			16 (50.0)	35 (53.0)	9 (56.3)
Latinx	54 (47.4)			16 (50.0)	31 (47.0)	7 (43.8)
Education, ≤ high school	63 (55.3)	36 (60.0)	27 (50.0)	21 (65.6)	33 (50.0)	9 (56.3)
Income, ≤ 200% federal poverty level	83 (74.1)^f^	47 (81.0)^f^	36 (66.7)	21 (65.6)	50 (76.9)^g^	12 (80.0)^g^
Marital status, never married	51 (44.7)	28 (46.7)	23 (42.6)	10 (31.3)	32 (48.5)	9 (56.3)
Menthol use, % yes	64 (56.1)	48 (80.0)	16 (29.6)	18 (56.3)	36 (54.5)	10 (62.5)
Number years smoking	17.3 (13.1)	19.3 (13.7)	15.0 (12.1)	13.6 (12.0)	16.8 (12.0)	26.7 (15.8)
Time to first cigarette ≤ 30 min	81 (71.1)	43 (71.7)	38 (70.4)	22 (68.8)	47 (71.2)	12 (75.0)
Days smoked/past 7^a^	6.8 (0.5)	6.8 (0.6)	6.9 (0.4)	6.8 (0.4)	6.9 (0.5)	6.8 (0.5)
Cigarettes per day/past 7^a^	12.4 (7.9)	12.3 (7.9)	12.5 (8.1)	11.2 (5.5)	12.4 (7.9)	14.7 (11.5)
Days used EC/past 7^a^	0.04 (0.30)	0.00 (0.00)	0.07 (0.43)	0.00 (0.00)	0.06 (0.39)	0.00 (0.00)
EC times on days used/past 7^a^	0.06 (0.51)	0.00 (0.00)	0.12 (0.74)	0.00 (0.00)	0.10 (0.67)	0.00 (0.00)
Past-year quit attempt, % yes^b^	37 (32.5)	18 (30.0)	19 (35.2)	13 (40.6)	19 (28.8)	5 (31.3)
Relied on friend/family support, % yes	8 (21.6)	4 (22.2)	4 (21.1)	1 (7.7)	5 (26.3)	2 (40.0)
Used counseling or other support resources, % yes^c^	11 (30.6)^g^	6 (35.3)^g^	5 (26.3)	2 (15.4)	8 (44.4)^g^	1 (20.0)
Used nicotine patch, gum, inhaler, nasal spray, lozenge or pill, % yes	7 (18.9)	2 (11.1)	5 (26.3)	2 (15.4)	3 (15.8)	2 (40.0)
Used Chantix (varenicline) or Zyban (Wellbutrin or bupropion), % yes	4 (10.8)	4 (22.2)	0 (0)	0 (0)	3 (15.8)	1 (20.0)
Used a different tobacco product, % yes^d^	6 (16.2)	0 (0)	6 (31.6)	2 (15.4)	4 (21.1)	0 (0)
History of chronic obstructive pulmonary disease, % yes	10 (8.8)^g^	7 (11.9)^g^	3 (5.6)	4 (12.9)^g^	4 (6.1)	2 (12.5)
History of asthma, % yes	29 (25.7)^g^	17 (28.8)^g^	12 (22.2)	7 (22.6)^g^	18 (27.3)	4 (25.0)
Mental health history, % any history^e^	72 (63.2)	40 (66.7)	32 (59.3)	21 (65.6)	40 (60.6)	11 (68.8)
History of substance abuse, % yes	58 (50.9)	29 (48.3)	29 (53.7)	15 (46.9)	32 (48.5)	11 (68.8)

Table displays baseline study sample characteristics of the EC group. Variables are reported for all, split by race/ethnicity, and split by tobacco use trajectory

^a^From 7-day timeline follow-back

^b^Denominator for quit attempt strategies

^c^Other support resources include a telephone help line or quit line, books, pamphlets, videos, a quit tobacco clinic, class, or support group, and internet or web-based programs

^d^Different tobacco products include e-cigarettes, traditional cigars, cigarillos, filtered cigars, pipe tobacco, hookah, snus pouches, smokeless tobacco (dip, chew, snuff), and dissolvable tobacco

^e^Self-reported history of depression, anxiety, PTSD, or schizophrenia

^f^Two values missing

^g^One value missing

### Facilitators of switching

#### What participants liked about using JUUL

Clean/smell was the leading reason JUUL was liked by both AA (25%) and Lx (21%) participants (e.g., “Nice not feeling like you smelled bad after, not having residue and ash all over”). Convenience was the second leading reason (22% AA; 16% Lx; e.g., “I liked that it was convenient. No lighters”) (Table 4). Additional themes liked about using JUUL identified by both AA and Lx participants included health, cost, taste, subjective effects, craving/cessation support, social, and “nothing.” There was one unique theme among Lx participants: use anywhere.

**Table 4:** Content analysis thematic results, by race/ethnicity and tobacco use trajectory

	African American/Kansas City				Latinx/San Diego			
	All N (%)	Exclusive JUUL Use N (%)	Dual JUUL and Cigarette Use N (%)	Continued Cigarette Use N (%)	All N (%)	Exclusive JUUL use N (%)	Dual JUUL and Cigarette Use N (%)	Continued Cigarette Use N (%)
*Liked*	*n* items = 99	*n* items = 30	*n* items = 57	*n* items = 12	*n* items = 82	*n* items = 25	*n* items = 46	*n* items = 11
Clean/smell	25 (25.3%)	10 (33.3%)	13 (22.8%)	2 (16.7%)	17 (20.7%)	8 (32.0%)	6 (13.0%)	3 (27.3%)
Convenience	22 (22.2%)	5 (16.7%)	14 (24.6%)	3 (25.0%)	13 (15.9%)	4 (16.0%)	8 (17.4%)	1 (9.1%)
Health	13 (13.1%)	8 (26.7%)	5 (8.8%)	0 (0.0%)	8 (9.8%)	3 (12.0%)	4 (8.7%)	1 (9.1%)
Cost	13 (13.1%)	1 (3.3%)	10 (17.5%)	2 (16.7%)	3 (3.7%)	1 (4.0%)	1 (2.2%)	1 (9.1%)
Taste	11 (11.1%)	3 (10.0%)	6 (10.5%)	2 (16.7%)	11 (13.4%)	2 (8.0%)	8 (17.4%)	1 (9.1%)
Subjective effects	6 (6.1%)	1 (3.3%)	5 (8.8%)	0 (0.0%)	5 (6.1%)	1 (4.0%)	4 (8.7%)	0 (0.0%)
Cravings/cessation	5 (5.1%)	2 (6.7%)	3 (5.3%)	0 (0.0%)	7 (8.5%)	3 (12.0%)	2 (4.3%)	2 (18.2%)
Nothing	2 (2.0%)	0 (0.0%)	0 (0.0%)	2 (16.7%)	1 (1.2%)	0 (0.0%)	0 (0.0%)	1 (9.1%)
Social	2 (2.0%)	0 (0.0%)	1 (1.8%)	1 (8.3%)	7 (8.5%)	0 (0.0%)	7 (15.2%)	0 (0.0%)
Use anywhere^a^					10 (12.2%)	3 (12.0%)	6 (13.0%)	1 (9.1%)
*Helped*	*n* items = 63	*n* items = 16	*n* items = 38	*n* items = 9	*n* items = 66	*n* items = 22	*n* items = 37	*n* items = 7
Self-regulation/motivation	20 (31.7%)	6 (37.5%)	12 (31.6%)	2 (22.2%)	14 (21.2%)	5 (22.7%)	8 (21.6%)	1 (14.3%)
Nothing/NA	17 (27.0%)	1 (6.3%)	10 (26.3%)	6 (66.7%)	2 (3.0%)	0 (0.0%)	1 (2.7%)	1 (14.3%)
Cravings/cessation	4 (6.3%)	2 (12.5%)	2 (5.3%)	0 (0.0%)	4 (6.1%)	2 (9.1%)	2 (5.4%)	0 (0.0%)
Health	3 (4.8%)	0 (0.0%)	2 (5.3%)	1 (11.1%)	7 (10.6%)	1 (4.5%)	5 (13.5%)	1 (14.3%)
Convenience	2 (3.2%)	1 (6.3%)	1 (2.6%)	0 (0.0%)	9 (13.6%)	4 (18.2%)	4 (10.8%)	1 (14.3%)
Social	1 (1.6%)	0 (0.0%)	1 (2.6%)	0 (0.0%	5 (7.6%)	3 (13.6%)	1 (2.7%)	1 (14.3%)
Cost	1 (1.6%)	0 (0.0%)	1 (2.6%)	0 (0.0%)	6 (9.1%)	4 (18.2%)	1 (2.7%)	1 (14.3%)
Program support	1 (1.6%)	1 (6.3%)	0 (0.0%)	0 (0.0%)	8 (12.1%)	1 (4.5%)	7 (18.9%)	0 (0.0%)
Practice^b^	13 (20.6%)	4 (25.0%)	9 (23.7%)	0 (0.0%)				
Subjective experience^b^	1 (1.6%)	1 (6.3%)	0 (0.0%)	0 (0.0%)				
Flavor^a^					7 (10.6%)	1 (4.5%)	5 (13.5%)	1 (14.3%)
Smell^a^					2 (3.0%)	1 (4.5%)	1 (2.7%)	0 (0.0%)
Other^a^					2 (3.0%)	0 (0.0%)	2 (5.4%)	0 (0.0%)
*Not liked*	*n* items = 67	*n* items = 17	*n* items = 41	*n* items = 9	*n* items = 58	*n* items = 16	*n* items = 35	*n* items = 7
Nothing	23 (34.3%)	9 (52.9%)	14 (34.1%)	0 (0.0%)	17 (29.3%)	8 (50.0%)	8 (22.9%)	1 (14.3%)
Side effects (coughing/harshness)	23 (34.3%)	3 (17.6%)	14 (34.1%)	6 (66.7%)	7 (12.1%)	2 (12.5%)	2 (5.7%)	3 (42.9%)
Mechanical issues—pods	6 (9.0%)	1 (5.9%)	4 (9.8%)	1 (11.1%)	6 (10.3%)	1 (6.3%)	5 (14.3%)	0 (0.0%)
Inconvenience^b^	7 (10.4%)	1 (5.9%)	5 (12.2%)	1 (11.1%)				
Shape^b^	4 (6.0%)	2 (11.8%)	2 (4.9%)	0 (0.0%)				
Taste^b^	3 (4.5%)	0 (0.0%)	2 (4.9%)	1 (11.1%)				
Learning to use^b^	1 (1.5%)	1 (5.9%)	0 (0.0%)	0 (0.0%)				
User issues^a^					8 (13.8%)	1 (6.3%)	7 (20.0%)	0 (0.0%)
Mechanical issues—battery^a^					8 (13.8%)	3 (18.8%)	4 (11.4%)	1 (14.3%)
Comparability^a^					7 (12.1%)	0 (0.0%)	6 (17.1%)	1 (14.3%)
Nicotine exposure^a^					4 (6.9%)	1 (6.3%)	2 (5.7%)	1 (14.3%)
Cost^a^					1 (1.7%)	0 (0.0%)	1 (2.9%)	0 (0.0%)
*Difficult*	*n* items = 62	*n* items = 17	*n* items = 36	*n* items = 9	*n* items = 55	*n* items = 16	*n* items = 32	*n* items = 7
Side effects	27 (43.5%)	3 (17.6%)	18 (50.0%)	6 (66.7%)	5 (9.1%)	1 (6.3%)	2 (6.3%)	2 (28.6%)
Nothing	24 (38.7%)	11 (64.7%)	11 (30.6%)	2 (22.2%)	24 (43.6%)	9 (56.3%)	13 (40.6%)	2 (28.6%)
Comparability	2 (3.2%)	1 (5.9%)	1 (2.8%)	0 (0.0%)	8 (14.5%)	0 (0.0%)	7 (21.9%)	1 (14.3%)
Other	1 (1.6%)	0 (0.0%)	1 (2.8%)	0 (0.0%)	2 (3.6%)	1 (6.3%)	1 (3.1%)	0 (0.0%)
Taste^b^	2 (3.2%)	0 (0.0%)	1 (2.8%)	1 (11.1%)				
Readiness to quit^b^	2 (3.2%)	0 (0.0%)	2 (5.6%)	0 (0.0%)				
Strength/nicotine intensity^b^	2 (3.2%)	1 (5.9%)	1 (2.8%)	0 (0.0%)				
Mechanical issues—pods^b^	1 (1.6%)	0 (0.0%)	1 (2.8%)	0 (0.0%)				
Learning to use^b^	1 (1.6%)	1 (5.9%)	0 (0.0%)	0 (0.0%)				
User issues^a^					6 (10.9%)	1 (6.3%)	3 (9.4%)	2 (28.6%)
Craving for cigarettes^a^					6 (10.9%)	2 (12.5%)	4 (12.5%)	0 (0.0%)
Social^a^					3 (5.5%)	2 (12.5%)	1 (3.1%)	0 (0.0%)
Cost^a^					1 (1.8%)	0 (0.0%)	1 (3.1%)	0 (0.0%)

Table displays content analysis thematic results for responses to what participants liked about using ECs, what helped participants with switching to ECs, what participants did not like about using ECs, and what made switching to ECs difficult. Results are reported by race/ethnicity and tobacco use trajectory

^a^Theme is unique to Lx/San Diego sample

^b^Theme is unique to AA/Kansas City sample

By week 6 use trajectory, the leading aspects liked about using JUUL among those who exclusively used JUUL were clean/smell (33%) and health-related reasons (20%) (Table 5; Additional file 1 those who dually used JUUL and cigarettes cited convenience (21%) and clean/smell (18%). Those who continued smoking reported clean/smell most frequently (22%), followed by convenience (17%).

**Table 5:** Content analysis thematic results, by tobacco use trajectory

	All N (%)	Exclusive JUUL use N (%)	Dual JUUL and cigarette use N (%)	Continued cigarette use N (%)
*Liked*	*n* items = 181	*n* items = 55	*n* items = 103	*n* items = 23
Clean/smell	42 (23.2%)	18 (32.7%)	19 (18.4%)	5 (21.7%)
Convenience	35 (19.3%)	9 (16.4%)	22 (21.4%)	4 (17.4%)
Health	21 (11.6%)	11 (20.0%)	9 (8.7%)	1 (4.3%)
Cost	16 (8.8%)	2 (3.6%)	11 (10.7%)	3 (13.0%)
Taste	22 (12.2%)	5 (9.1%)	14 (13.6%)	3 (13.0%)
Subjective effects	11 (6.1%)	2 (3.6%)	9 (8.7%)	0 (0.0%)
Cravings/cessation	12 (6.6%)	5 (9.1%)	5 (4.9%)	2 (8.7%)
Nothing	3 (1.7%)	0 (0.0%)	0 (0.0%)	3 (13.0%)
Social	9 (5.0%)	0 (0.0%)	8 (7.8%)	1 (4.3%)
Use anywhere^a^	10 (5.5%)	3 (5.5%)	6 (5.8%)	1 (4.3%)
*Helped*	*n* items = 129	*n* items = 38	*n* items = 75	*n* items = 16
Self-regulation/motivation	34 (26.4%)	11 (28.9%)	20 (26.7%)	3 (18.8%)
Nothing/NA	19 (14.7%)	1 (2.6%)	11 (14.7%)	7 (43.8%)
Cravings/cessation	8 (6.2%)	4 (10.5%)	4 (5.3%)	0 (0.0%)
Health	10 (7.8%)	1 (2.6%)	7 (9.3%)	2 (12.5%)
Convenience	11 (8.5%)	5 (13.2%)	5 (6.7%)	1 (6.3%)
Social	6 (4.7%)	3 (7.9%)	2 (2.7%)	1 (6.3%
Cost	7 (5.4%)	4 (10.5%)	2 (2.7%)	1 (6.3%)
Program support	9 (7.0%)	2 (5.3%)	7 (9.3%)	0 (0.0%)
Practice^b^	13 (10.1%)	4 (10.5%)	9 (12.0%)	0 (0.0%)
Subjective experience^b^	1 (0.8%)	1 (2.6%)	0 (0.0%)	0 (0.0%)
Flavor^a^	7 (5.4%)	1 (2.6%)	5 (6.7%)	1 (6.3%)
Smell^a^	2 (1.6%)	1 (2.6%)	1 (1.3%)	0 (0.0%)
Other^a^	2 (1.6%)	0 (0.0%)	2 (2.7%)	0 (0.0%)
*Not liked*	*n* items = 125	*n* items = 33	*n* items = 76	*n* items = 16
Nothing	40 (32.0%)	17 (51.5%)	22 (28.9%)	1 (6.3%)
Side effects (coughing/harshness)	30 (24.0%)	5 (15.2%)	16 (21.1%)	9 (56.3%)
Mechanical issues—pods	12 (9.6%)	2 (6.1%)	9 (11.8%)	1 (6.3%)
Inconvenience^b^	7 (5.6%)	1 (3.0%)	5 (6.6%)	1 (6.3%)
Shape^b^	4 (3.2%)	2 (6.1%)	2 (2.6%)	0 (0.0%)
Taste^b^	3 (2.4%)	0 (0.0%)	2 (2.6%)	1 (6.3%)
Learning to use^b^	1 (0.8%)	1 (3.0%)	0 (0.0%)	0 (0.0%)
User issues^a^	8 (6.4%)	1 (3.0%)	7 (9.2%)	0 (0.0%)
Mechanical issues—battery^a^	8 (6.4%)	3 (9.1%)	4 (5.3%)	1 (6.3%)
Comparability^a^	7 (5.6%)	0 (0.0%)	6 (7.9%)	1 (6.3%)
Nicotine exposure^a^	4 (3.2%)	1 (3.0%)	2 (2.6%)	1 (6.3%)
Cost^a^	1 (0.8%)	0 (0.0%)	1 (1.3%)	0 (0.0%)
*Difficult*	*n* items = 117	*n* items = 33	*n* items = 68	*n* items = 16
Side effects	32 (27.4%)	4 (12.1%)	20 (29.4%)	8 (50.0%)
Nothing	48 (41.0%)	20 (60.6%)	24 (35.3%)	4 (25.0%)
Comparability	10 (8.5%)	1 (3.0%)	8 (11.8%)	1 (6.3%)
Other	3 (2.6%)	1 (3.0%)	2 (2.9%)	0 (0.0%)
Taste^b^	2 (1.7%)	0 (0.0%)	1 (1.5%)	1 (6.3%)
Readiness to quit^b^	2 (1.7%)	0 (0.0%)	2 (2.9%)	0 (0.0%)
Strength/nicotine intensity^b^	2 (1.7%)	1 (3.0%)	1 (1.5%)	0 (0.0%)
Mechanical issues—pods^b^	1 (0.9%)	0 (0.0%)	1 (1.5%)	0 (0.0%)
Learning to use^b^	1 (0.9%)	1 (3.0%)	0 (0.0%)	0 (0.0%)
User issues^a^	6 (5.1%)	1 (3.0%)	3 (4.4%)	2 (12.5%)
Craving for Cigarettes^a^	6 (5.1%)	2 (6.1%)	4 (5.9%)	0 (0.0%)
Social^a^	3 (2.6%)	2 (6.1%)	1 (1.5%)	0 (0.0%)
Cost^a^	1 (0.9%)	0 (0.0%)	1 (1.5%)	0 (0.0%)

Table displays content analysis thematic results for responses to what participants liked about using ECs, what helped participants with switching to ECs, what participants did not like about using ECs, and what made switching to ECs difficult. Results are reported for all and by tobacco use trajectory

^a^Theme is unique to Latinx/San Diego sample

^b^Theme is unique to African American/Kansas City sample

#### What helped with switching

Self-driven factors such as motivation was the most reported factor that helped with switching among both AA (32%) and Lx (21%) participants (e.g., “Just sticking with it and not giving up;” “Self-determination”) (Table 4). This theme was named “self-regulation/motivation.” Nothing was the second most common response among AA participants (27%). Additional themes identified by both AA and Lx participants included craving/cessation support, health, convenience, social, cost, program support, and “nothing.” Unique themes among AA participants included practice and subjective experience. Unique themes among Lx participants included flavor, smell, and other.

By week 6 use trajectory, the leading aspect that helped with switching to JUUL among those who exclusively switched was self-regulation/motivation (29%), followed by convenience (13%) (Table 5; Additional file 2). Among those who dually used JUUL and cigarettes, the top factor was also self-regulation/motivation (27%), followed by nothing (15%). For those who continued smoking, the leading factor was nothing (44%) followed by health (13%).

### Barriers to switching

#### What participants disliked about using JUUL

“Nothing” was the most reported response to what participants disliked about using JUUL among both AA (34%) and Lx (29%) participants (Table 4). The second leading reason was side effects such as coughing and harshness among AA participants (34%; e.g., “Coughing/harshness in throat”), while side effects were cited by Lx participants less often (12%). Additional aspects of JUUL disliked by both AA and Lx participants included mechanical issues with pods. Several issues described only by Lx participants included user issues, mechanical issues with battery, comparability to cigarettes, nicotine exposure, and cost. Issues described only by AA participants included inconvenience, shape, taste, and learning to use.

Among participants who exclusively switched to JUUL, “nothing” was the most frequent response (52%) and side effects was the second most frequent response (15%) (Table 5; Additional file 3). Among participants who dually used JUUL and cigarettes, “nothing” was also the most common response (29%) and side effects was the second most common response (21%). Among those who continued smoking, side effects were the most frequent response (56%).

#### What made switching difficult

The most common response to the question of what made switching to JUUL difficult was side effects among AA participants (44%; e.g., “Harshness, coughing”) and nothing among Lx participants (44%) (Table 4). Side effects were a less common issue cited by Lx participants (9.1%). Among AA participants, nothing was the second most common response (39%). Other responses among both AA and Lx participants included comparability to cigarettes and other. Issues uniquely coded among AA participants included taste; readiness to quit; strength/nicotine intensity; mechanical issues with pods, and learning to use. Issues uniquely coded among Lx participants included user issues; craving for cigarettes; social; and cost.

Among those who exclusively switched to JUUL, the most frequent answer was “nothing” (61%), followed by side effects (12%) (Table 5; Additional file 4). Among those who dually used JUUL and cigarettes, the most common response was “nothing” (35%), followed by side effects (29%). Among those who continued smoking, side effects were the most common issue (50%), followed by “nothing” (25%).

### Benefit/barrier ratio

The majority of both AA (78%) and Lx (90%) participants reported that the benefits of using JUUL outweighed barriers or concerns (Additional file 5). By JUUL use trajectory, the majority of individuals who exclusively switched to JUUL (94%), and dually used JUUL and cigarettes (86%) reported that the benefits of using JUUL outweighed barriers or concerns (Additional file 5). Most people who continued smoking reported that the benefits did not outweigh the barriers or concerns (58%) and another 42% reported that the benefits outweighed the barriers.

Among AA participants, the benefit to barrier ratio was incremental to rate of switching, with 94% of those who exclusively used JUUL reporting benefits outweighed barriers, compared to 77% of those who dually used and 50% of those who continued smoking. Among Lx participants, the benefit to barrier ratio was similar for exclusive JUUL use (93%) and dual use (97%). In contrast, more than half of those who continued smoking reported that the benefits do not outweigh barriers or concerns (75%).

### Future intentions toward continued use of ECs

The majority of AA (83%) and Lx (94%) participants said they would continue JUUL to replace cigarettes. The most common reasons for intention to continue using JUUL among AA participants were cigarette cessation (48%) and health (20%) (Additional file 6). The top reasons among Lx participants were also health (41%) and cigarette cessation (21%).

By week 6 use trajectory, there was a higher percentage of exclusive JUUL use (94%) and dual use (94%) than continued smoking (50%) who said they would continue using JUUL to replace cigarettes. The leading reasons to continue were for cigarette cessation (33%) and health (31%) (Additional file 6). The relative frequency of these reasons varied slightly by JUUL use trajectory, with health (37%) more common than cigarette cessation (35%) among individuals who exclusively switched to JUUL, and cigarette cessation (32%) more common than health (30%) among people who dually used JUUL and cigarettes. Cigarette cessation was the leading reason among those who continued smoking (38%), followed by “other” (25%).

### Perception of JUUL compared to previous cigarette reduction methods

The most common response when comparing JUUL to previous cigarette reduction methods among participants was that JUUL worked better than other methods to reduce cigarettes (55%) (e.g., “It was really good, better than Chantix, patch, gum, lozenge”), followed by it being the only method tried (13%) (“This is my first attempt at quitting”) (Additional file 7).

By site/race/ethnicity, the most common perception of JUUL compared to previous cigarette reduction methods among AA participants was that JUUL worked better than other methods to quit cigarettes (72%) (Additional file 7). Among Lx participants, that JUUL worked better than other methods was also common (39%) as were themes that JUUL was the only method tried (19%), had more pleasant sensations (10%), and was most similar to cigarettes (9%).

Responses were similar among those who exclusively switched to JUUL, with 54% reporting that it worked better than other methods and 16% stating it was the only method tried. Individuals who dually used JUUL and cigarettes had higher rates of reporting JUUL was better than other methods to quit cigarettes (63%). The most common response among those who continued smoking was that JUUL was worse than previous cigarette reduction methods (33%), followed by an equal proportion of worked better (22%) and that JUUL was the only method tried (22%).

## Discussion

The majority of AA and Lx participants reported a satisfactory experience using nicotine salt pod system (NSPS) ECs. Most reported that the benefits of using JUUL outweighed barriers or concerns and that they would continue using JUUL to replace cigarettes. The most common reasons for intending to continue using JUUL were cigarette cessation and health. NSPS ECs were viewed favorably compared to other cigarette reduction methods. Although ECs are not FDA-approved for cigarette cessation, over half of our participants stated that JUUL worked better than other methods to quit cigarettes. This echoed results found among people who smoked in lab studies, in which NSPS ECs provided greater satisfaction and liking than nicotine gum [24]. Simulation of smoking micro-behaviors such as hand to mouth movement and tactile sensations are one explanation for perceived superiority of ECs to pharmacological support [25].

The perception that JUUL worked better than previous cessation methods used was higher among our AA participants (72%). A majority of Lx participants also reported that JUUL worked better than previous cessation methods they had used (39%). A higher proportion of Lx participants said JUUL was the only method they had tried (19%). Less experience with cessation among our Lx participants may be an artifact of their younger age relative to our AA participants. Lx participants also cited a greater variety of reasons for their favorable comparison of JUUL to previous cessation methods they had used, including that JUUL gave them more pleasant sensations (10%) and JUUL was more similar to cigarettes (9%).

Our results generally contrast with a previous study of AA adults switching from smoking to first generation ECs which found that the majority (73%) reported an unsatisfactory experience [14]. One of the most important distinguishing characteristics between first generation cigalikes and fourth generation NSPS ECs is the nicotine salt formulation which provides more efficient nicotine delivery [20, 26]. Adequate nicotine replacement from NSPS ECs may have contributed to high rates of cigarette reduction in the parent study, including 28% who completely eliminated cigarettes [19].

The aspect of JUUL that participants most frequently cited as liking were the lack of smell and sense of cleanness, compared to smoking. Lack of smell was also a common reason for using ECs among AA and Lx adults in a US population-based study (69% AA; 73% Lx) [11]. The second aspect of JUUL that our participants frequently cited liking was convenience, which referred to aspects of size and not needing a lighter. In population-based surveys, being able to vape in places where smoking is not allowed was a leading reason for using ECs (84% AA; 78% Lx) [11]. While being able to use JUUL in more places is one aspect of convenience, our content analysis revealed that convenience encompassed additional practical considerations.

While the experience of using NSPS ECs was positive for the majority of participants, the benefits to barriers ratio of using JUUL was incremental to JUUL use trajectory: 94% exclusive JUUL use, 86% dual JUUL and cigarette use, and 42% continued cigarettes. The leading aspect of JUUL disliked and top barrier to using JUUL was coughing and harshness, which was more common among those who dually used JUUL and cigarettes and those who continued to smoke. This adverse effect was also reported more frequently by AA than Lx adults. It is possible that population-based characteristics in puffing behavior may have contributed to a greater experience of coughing and harshness with JUUL among our AA adults. There is evidence that AA adults take longer puffs [27] and extract more nicotine per cigarette smoked, relative to whites [28]. Depth of inhalation may have impacted the experience of JUUL as harsh and induced coughing. More research is needed in this area.

Self-regulation/motivation was the most commonly reported factor that helped with switching to JUUL. This suggests that interventions which boost self-efficacy and motivation could be valuable in supporting people who smoke to transition from cigarettes to NSPS ECs. Such interventions have proven useful for smoking cessation [29]. Additionally, practice was cited as a factor that helped with switching by AA participants, suggesting that coaching and accessible information about correct usage of JUUL could be useful in promoting transition from cigarettes to NSPS ECs. AA individuals who smoke may particularly benefit from instruction in modifying NSPS EC puffing relative to cigarettes, and practice may mitigate their experience of ECs as harsh. Future research is needed in this area.

A strength of the study was provision of four flavors which adds to ecological validity given the importance of taste to adults who smoke when using ECs for harm reduction [13]. Taste/flavor was a minor facilitator or barrier to switching, and it is possible that bans of flavored ECs could undermine the success of some adults who smoke using ECs for harm reduction. Banning menthol ECs could be particularly discouraging of switching among AA adults who predominantly smoke menthol cigarettes [14] and who we found were likely to seek a menthol flavored EC substitute [19]. Lack of access to an acceptable substitute could further widen tobacco-related health disparities for members of under-represented ethnic minority groups [30].

Study limitations include only one NSPS EC being tested (JUUL); results may not be generalizable to other brands of these devices (e.g., NJOY, VUSE) or to older, non-NSPS systems. Additionally, some flavors used in the study are no longer available on the U.S. market and it is unknown how study results would be impacted by restriction in flavors. An important future research avenue would be to evaluate the role of flavors in user experience, particularly among those who smoked menthol cigarettes. Furthermore, enrollment of AA people was limited to Kansas City, MO and enrollment of Lx people was limited to San Diego, CA. While there were no site interactions on study outcomes [19], generalizability would be improved by a more comprehensive sampling strategy.

## Conclusions

There is an urgent need to provide avenues to address tobacco-related health disparities [31]. Lessening harm for people who smoke among the largest racial/ethnic minority groups in the US is a top priority [9, 10]. Our research shows that NSPS ECs demonstrate promise for cigarette reduction among AA and Lx adults.

## Supplementary Information

**Additional file 1. **Frequencies of what liked about using JUUL by week 6 trajectory. Displays the frequencies of what participants liked about using JUUL by week 6 trajectory (exclusive JUUL use, dual JUUL and cigarette use, and continued cigarette use). Panel A shows the full sample, and Panels B and C show results split by the African American sample and the Latinx sample, respectively.

**Additional file 2**. Frequencies of what helped with switching to JUUL by week 6 trajectory. Displays the frequencies of what helped participants with switching to JUUL by week 6 JUUL trajectory (exclusive JUUL use, dual JUUL and cigarette use, and continued cigarette use). Panel A shows the full sample, and Panels B and C show results split by the African American sample and the Latinx sample, respectively.

**Additional file 3. **Frequencies of what did not like about using JUUL by week 6 trajectory. Displays the frequencies of what participants did not like about using JUUL by week 6 trajectory (exclusive JUUL use, dual JUUL and cigarette use, and continued cigarette use). Panel A shows the full sample, and Panels B and C show results split by the African American sample and the Latinx sample, respectively.

**Additional file 4.** Frequencies of what made switching to JUUL difficult by week 6 trajectory. Displays frequencies of what made switching to JUUL difficult by week 6 trajectory (exclusive JUUL use, dual JUUL and cigarette use, and continued cigarette use). Panel A shows the full sample, and Panels B and C show results split by the African American sample and the Latinx sample, respectively.

**Additional file 5. **Benefit to barrier ratio. Displays the benefit to barrier ratio responses of “Yes, benefits outweigh barriers or concerns”, “benefits and barriers are equal”, and “No, benefits do not outweigh barriers or concerns” split by week 6 trajectory (exclusive JUUL use, dual JUUL and cigarette use, and continued cigarette use). Panel A shows the full sample, and Panels B and C show results split by the African American sample and the Latinx sample, respectively.

**Additional file 6. **Frequencies of why continue using JUUL. Displays frequencies of why participants would continue using JUUL to replace cigarettes split by week 6 trajectory (exclusive JUUL use, dual JUUL and cigarette use, and continued cigarette use). Panel A shows the full sample, and Panels B and C show results split by the African American sample and the Latinx sample, respectively.

**Additional file 7.** Frequencies of comparability of JUUL to other methods to quit cigarettes. Displays frequencies of how JUUL compares to previous cigarette reduction methods split by week 6 trajectory (exclusive JUUL use, dual JUUL and cigarette use, and continued cigarette use). Panel A shows the full sample, and Panels B and C show results split by the African American sample and the Latinx sample, respectively.

## Data Availability

The datasets used and/or analyzed during the current study are available from the corresponding author on reasonable request.
