# Supplementary material for: “It is the One Thing that has Worked”: facilitators and barriers to switching to nicotine salt pod system e-cigarettes among African American and Latinx people who smoke: a content analysis
Source: Harm Reduct J. 2021 Sep 16;18:98. doi: 10.1186/s12954-021-00543-y (PMC8447685; doi:10.1186/s12954-021-00543-y)

Additional file 2. Frequencies of what helped with switching to JUUL by week 6 trajectory

Panel A. Full sample

*Notes.* The “Practice” and “Subjective Experience” themes are unique to the African American/Kansas City sample. The “Flavor”, “Smell”, and “Other” themes are unique to the Latinx/San Diego sample.

Panel B. African American sample


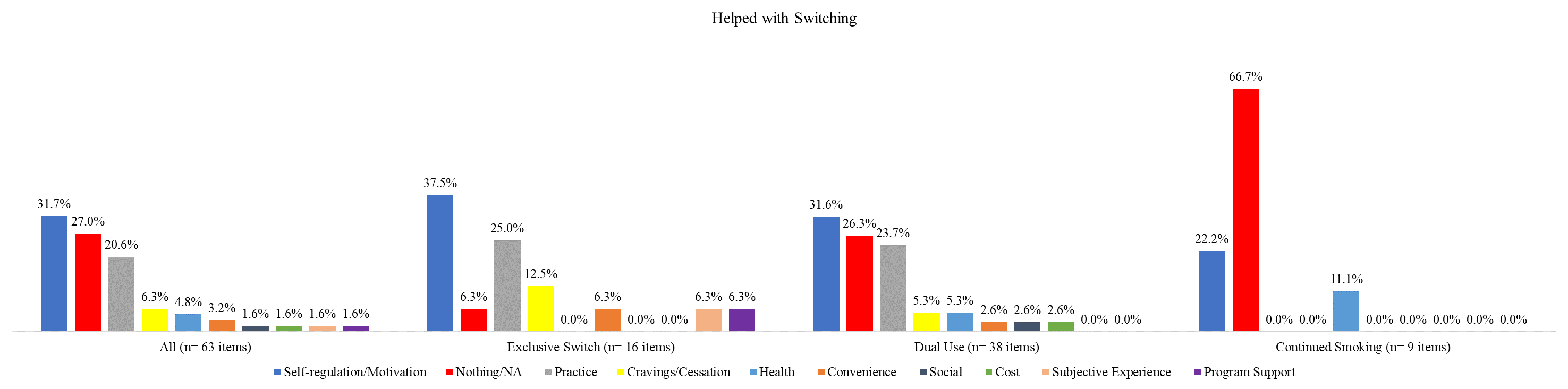


Panel C. Latinx sample


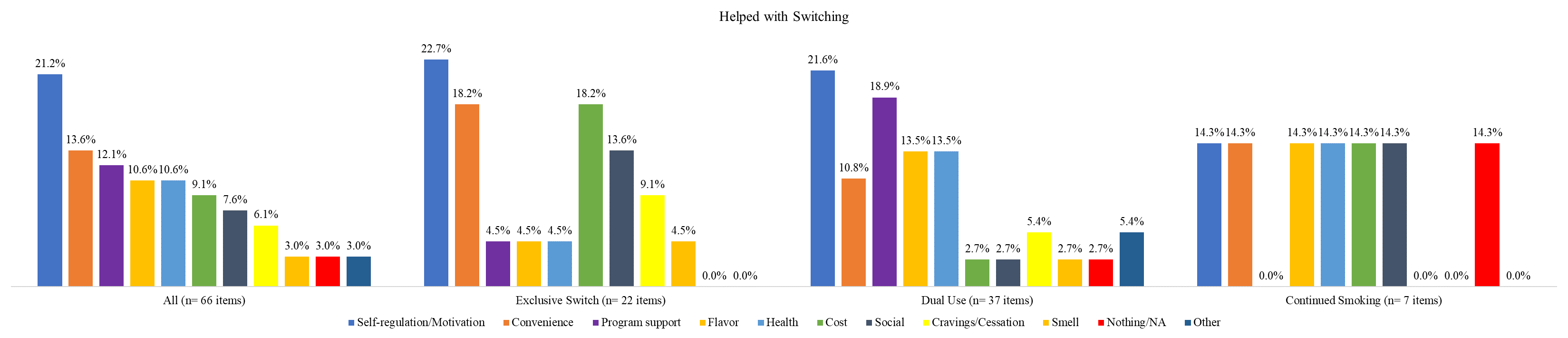

Supplement: Supplementary file 2 — Additional file 2. Frequencies of what helped with switching to JUUL by week 6 trajectory. Displays the frequencies of what helped participants with switching to JUUL by week 6 JUUL trajectory (exclusive JUUL use, dual JUUL and cigarette use, and continued cigarette use). Panel A shows the full sample, and Panels B and C show results split by the African American sample and the Latinx sample, respectively. [file 12954_2021_543_MOESM2_ESM.docx]
