# Supplementary material for: “It is the One Thing that has Worked”: facilitators and barriers to switching to nicotine salt pod system e-cigarettes among African American and Latinx people who smoke: a content analysis
Source: Harm Reduct J. 2021 Sep 16;18:98. doi: 10.1186/s12954-021-00543-y (PMC8447685; doi:10.1186/s12954-021-00543-y)

Additional file 4. Frequencies of what made switching to JUUL difficult by week 6 trajectory

Panel A. Full sample

*Notes.* The “Taste”, “Readiness to Quit”, “Strength/Nicotine Intensity”, “Mechanical Issues-Pods”, and “Learning to Use” themes are unique to the African American/Kansas City sample. The “User Issues”, “Cravings for Cigarettes”, “Social”, and “Cost” themes are unique to the Latinx/San Diego sample.

Panel B. African American sample


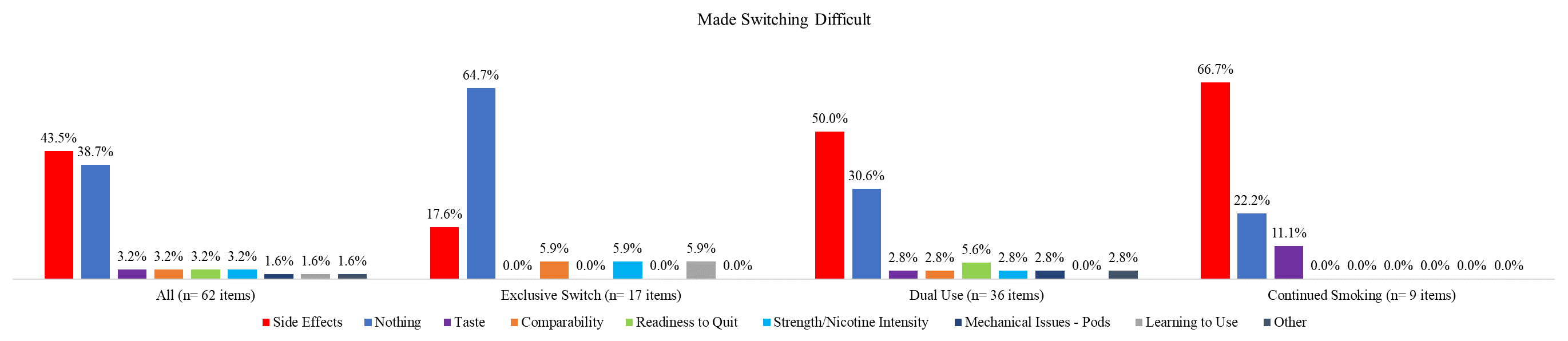


Panel C. Latinx sample
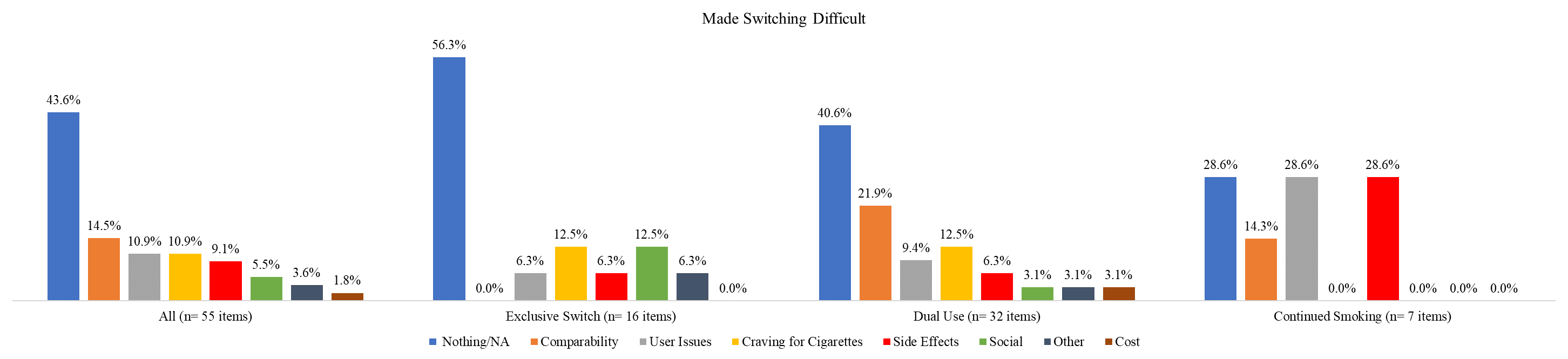

Supplement: Supplementary file 4 — Additional file 4. Frequencies of what made switching to JUUL difficult by week 6 trajectory. Displays frequencies of what made switching to JUUL difficult by week 6 trajectory (exclusive JUUL use, dual JUUL and cigarette use, and continued cigarette use). Panel A shows the full sample, and Panels B and C show results split by the African American sample and the Latinx sample, respectively. [file 12954_2021_543_MOESM4_ESM.docx]
