# Supplementary material for: “It is the One Thing that has Worked”: facilitators and barriers to switching to nicotine salt pod system e-cigarettes among African American and Latinx people who smoke: a content analysis
Source: Harm Reduct J. 2021 Sep 16;18:98. doi: 10.1186/s12954-021-00543-y (PMC8447685; doi:10.1186/s12954-021-00543-y)

Additional file 5. Benefit to barrier ratio

Panel A. All


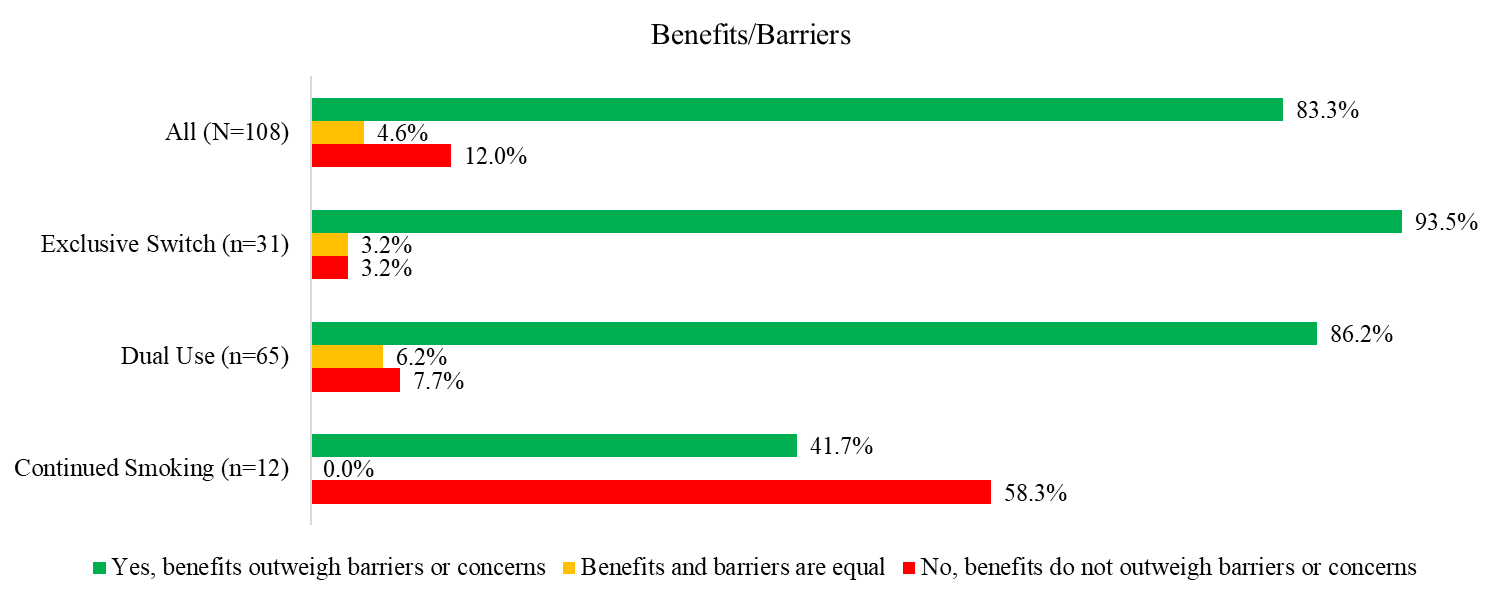


Panel B. African American sample


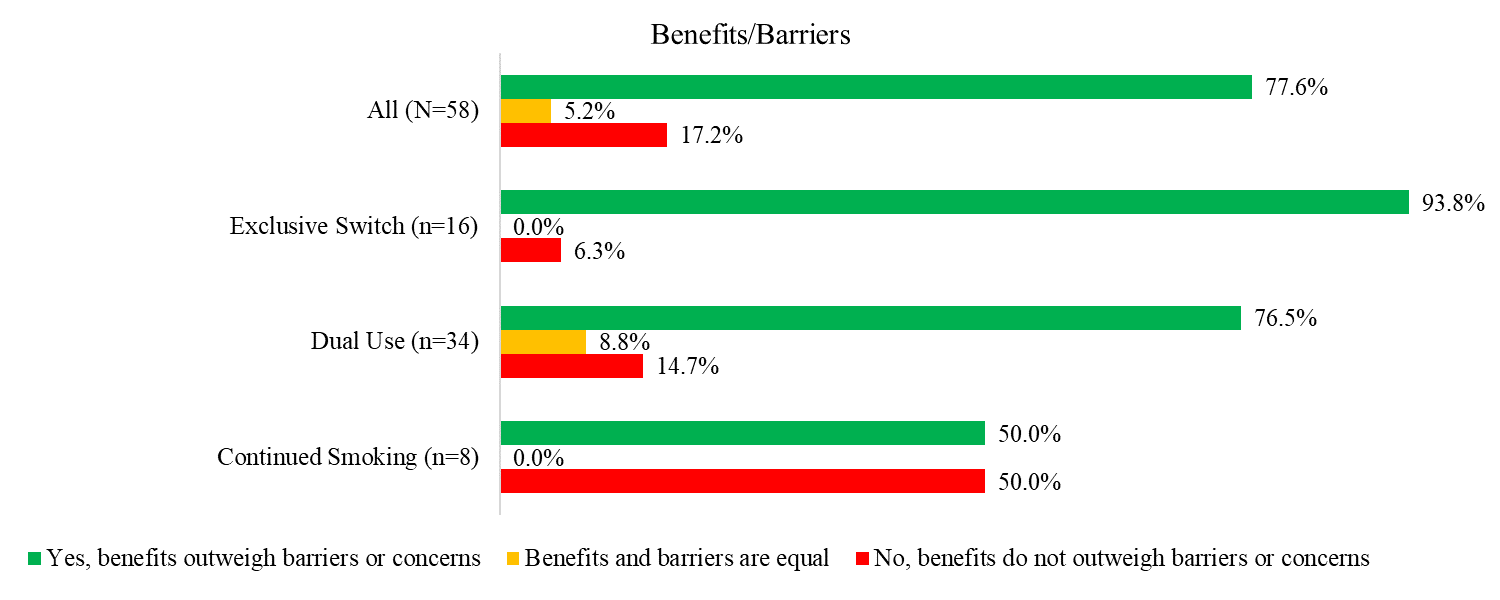


Panel C. Latinx sample


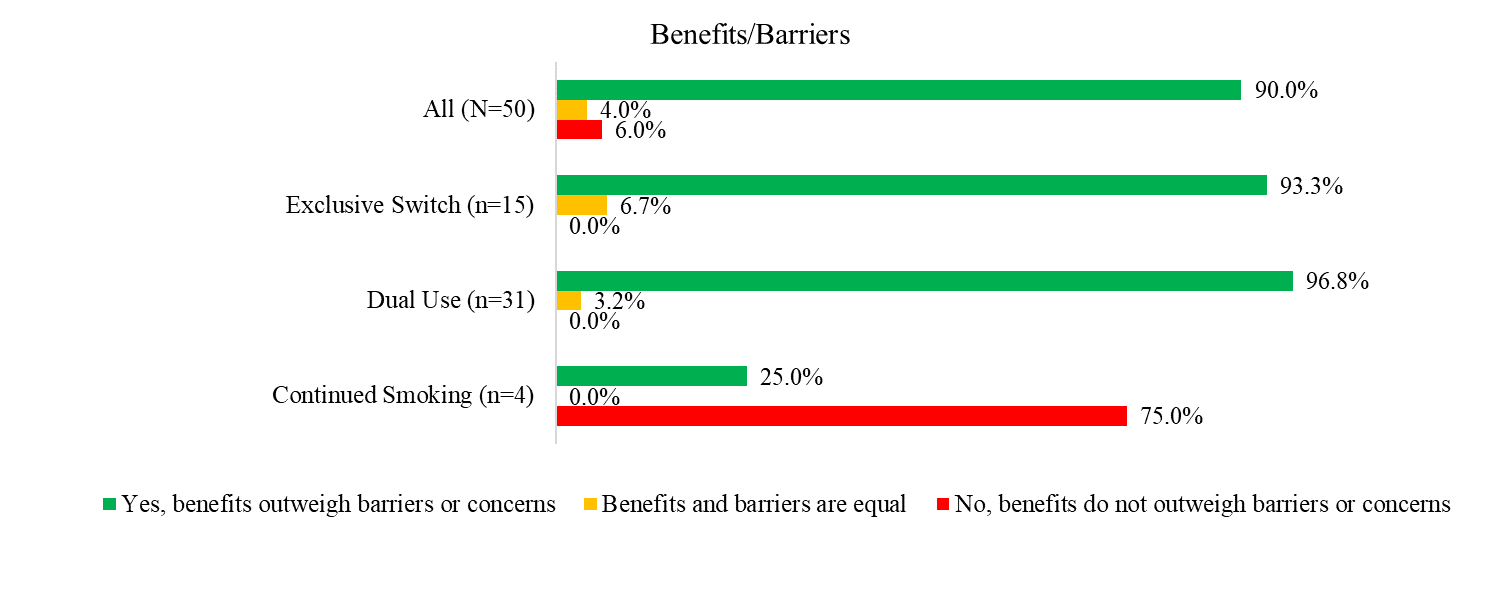

Supplement: Supplementary file 5 — Additional file 5. Benefit to barrier ratio. Displays the benefit to barrier ratio responses of “Yes, benefits outweigh barriers or concerns”, “benefits and barriers are equal”, and “No, benefits do not outweigh barriers or concerns” split by week 6 trajectory (exclusive JUUL use, dual JUUL and cigarette use, and continued cigarette use). Panel A shows the full sample, and Panels B and C show results split by the African American sample and the Latinx sample, respectively. [file 12954_2021_543_MOESM5_ESM.docx]
