# Supplementary material for: “It is the One Thing that has Worked”: facilitators and barriers to switching to nicotine salt pod system e-cigarettes among African American and Latinx people who smoke: a content analysis
Source: Harm Reduct J. 2021 Sep 16;18:98. doi: 10.1186/s12954-021-00543-y (PMC8447685; doi:10.1186/s12954-021-00543-y)

Additional file 6. Frequencies of why continue using JUUL

Panel A. All


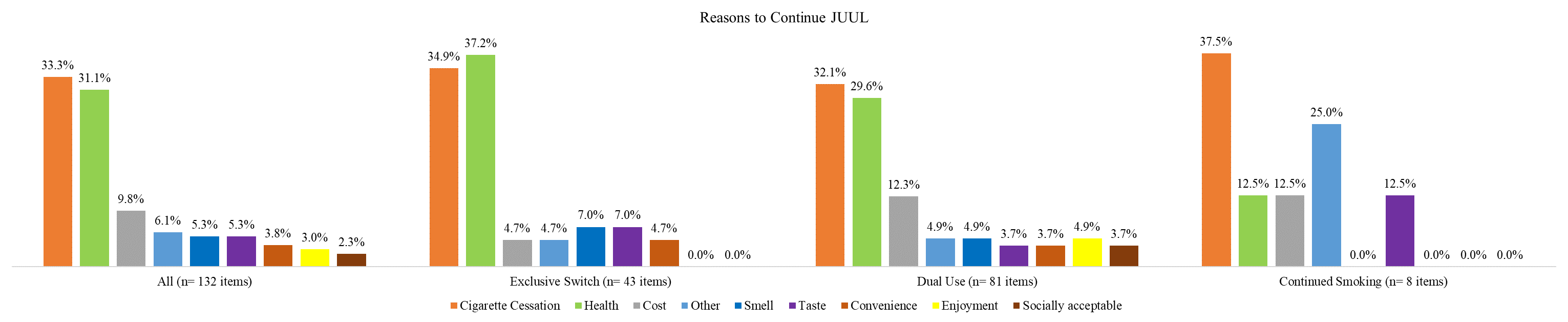


Panel B. African American sample


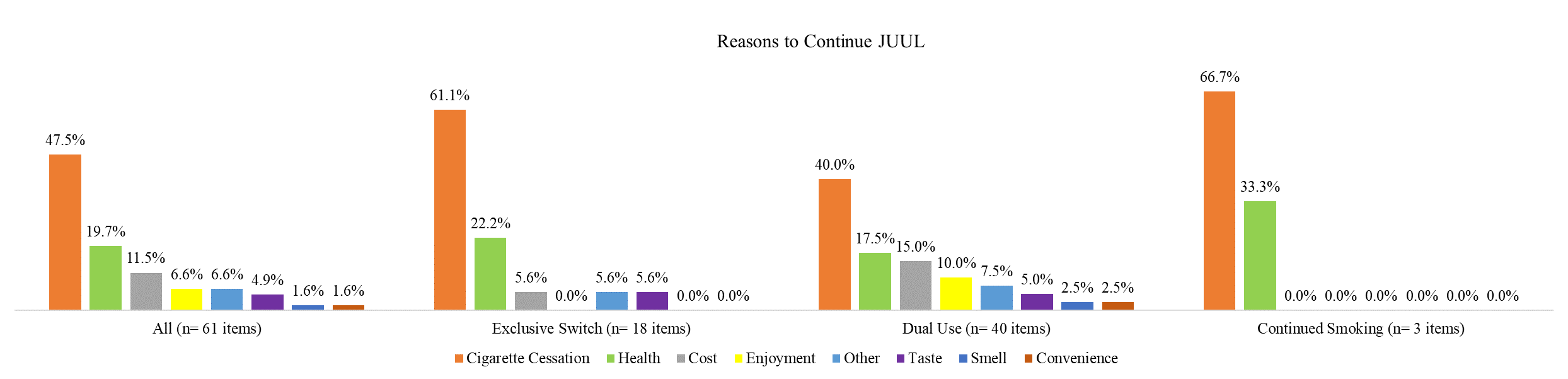


Panel C. Latinx sample


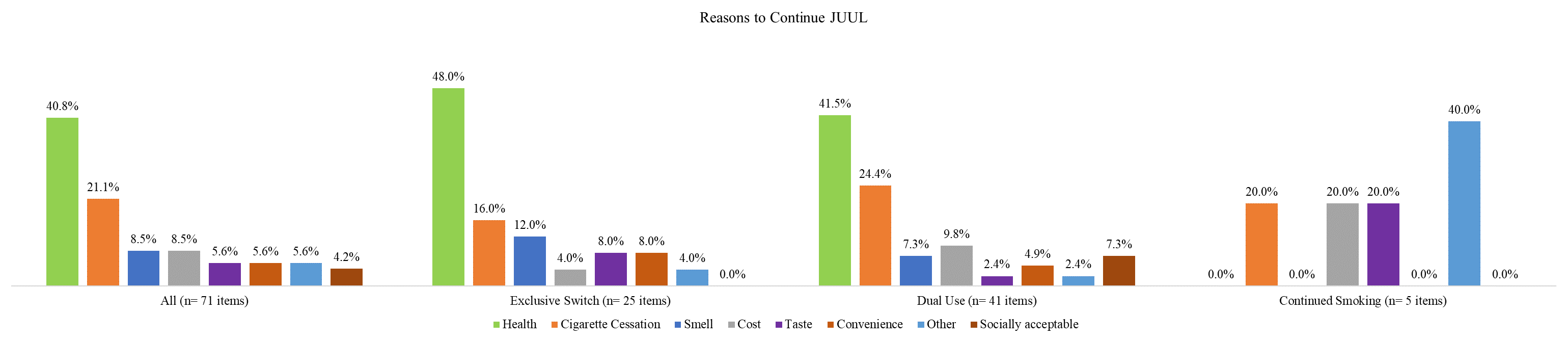

Supplement: Supplementary file 6 — Additional file 6. Frequencies of why continue using JUUL. Displays frequencies of why participants would continue using JUUL to replace cigarettes split by week 6 trajectory (exclusive JUUL use, dual JUUL and cigarette use, and continued cigarette use). Panel A shows the full sample, and Panels B and C show results split by the African American sample and the Latinx sample, respectively. [file 12954_2021_543_MOESM6_ESM.docx]
